# Transfer of Students

**Published:** 1898-05

**Authors:** 


### Transfer of Students.

It is becoming very common for students of dentistry to take one or two courses of their college education in one college and go to another for the remainder.

Perhaps different reasons will be found in different cases for such transfers. One is occasionally found in the fact that the college work will involve less expense at one place than at another, or a student sometimes imagines that he can obtain better training at one school than at another, and in some instances such transfer is made to save time and avoid more prolonged work and study. Occasionally a student will become offended with one or more of his teachers and will determine to secure new teachers.

There is always more or less objection to such changes.

There is usually more or less difference in the scheme of studies, and in the modes of teaching and in the direction of students, so that in going from one college to another it is always more or less difficult for a student to conform to the new order. He may not, and oftentimes does not, comprehend the methods of his new teachers. Such students are frequently an annoyance to teachers, and by no means helpful to the class they have entered.

The passage of students from one college to another oftentimes involves to a greater or lesser extent.

An undesirable student may pass along for a year or two without incurring the disapprobation of his teachers, and that, too, while it is known that he lacks in fitness for that which he is trying to do.

So that, unless there is some very palpable reason for changing from one school to another, it ought to be discouraged. It is far more satisfactory to the efficient teacher to carry his pupil through his entire course.

